# Prevalence of malocclusion in primary dentition in mainland China, 1988–2017: a systematic review and meta-analysis

**DOI:** 10.1038/s41598-018-22900-x

**Published:** 2018-03-16

**Authors:** Lu Shen, Fang He, Cai Zhang, Haofeng Jiang, Jinhua Wang

**Affiliations:** 1 College of Stomatology, Chongqing Medical University, Chongqing, 401147 China; 2 Chongqing Key Laboratory of Oral Diseases and Biomedical Sciences, Chongqing, 401147 China; 3 Chongqing Municipal Key Laboratory of Oral Biomedical Engineering of Higher Education, Chongqing, 401147 China; 4 School of Public Health and Management, Chongqing Medical University, Research Centre for Medicine and Social Development, Collaborative Innovation Centre of Social Risks Governance in Health, Chongqing Medical University, Chongqing, 400016 China

## Abstract

Malocclusion is a common oral disease affecting children with various reported prevalence rates. This meta-analysis aimed to determine the epidemiological characteristics of malocclusion among pre-schoolers in mainland China from 1988 to 2017. A total of 31 qualified papers describing 51,100 Chinese children aged 2–7 years were selected. The pooled malocclusion prevalence was 45.50% (95% confidence interval (CI): 38.08–52.81%) with 26.50% Class I (CI: 19.96–33.12%), 7.97% Class II (CI: 6.06–9.87%) and 12.60% Class III (CI: 9.45–15.68%) cases. The most common type of malocclusion was overbite (33.66%, CI: 27.66–39.67%), and the flush terminal type (47.10%, CI: 28.76–65.44%) was the most common in the terminal plane relationship. An increasing trend and wide variations across the country were observed. Additionally, there was no significant difference in malocclusion by gender (relative risk (RR) = 1.01, [0.96–1.06]) or urban/rural area (RR = 0.99, [0.82–1.20]). Although this study represents a narrow view of deciduous-dentition malocclusion in mainland China, the results provide sample evidence that can aid clinicians and policy makers towards early prevention and timely treatment.

## Introduction

Malocclusion is regarded as an irregularity of the teeth or a mal-relationship between the dental arches beyond the normal range^1^. The etiology of malocclusion is multifactorial, including genetic and environmental causes as well as harmful oral habits^2^. Malocclusion represents a developmental disorder of the craniofacial complex that affects the jaws, tongue and facial muscles^3^ and is known as one of the three major oral diseases that affect human oral function, aesthetics, social interactions and health-related quality of life^4,5^.

Previous cohort studies have indicated that malocclusion in primary dentition leads to malocclusion in permanent dentition^6,7^. If untreated, over time, malocclusion can vary from mild to severe, with varying impacts on aesthetics and/or function^8^. With the implementation of the “two children” policy in China, children’s malocclusion has become a major oral health issue of interest. Currently, there is an increasing trend in studies focusing on the early diagnosis and treatment of malocclusion in primary dentition.

The disease burden of malocclusion among pre-schoolers exhibits substantial variability worldwide, with prevalence rates ranging from 26.0% in India^9^ to 87.0% in Brazil^10^. Various provinces and cities in mainland China have conducted epidemiological surveys on deciduous-dentition malocclusion. Similarly, there seems to be a wide range of reported prevalence rates, from 13.15% in Ningxia^11^ to 83.94% in Shanghai^12^. The most recent and comprehensive study on deciduous-dentition malocclusion was undertaken by the Chinese Stomatological Association (CSA) in 2000, reporting a malocclusion prevalence of 51.84% in Chinese children^13^. However, the random sampling survey was performed in only 12 cities across China.

To the best of our knowledge, there is a lack of comprehensive and critical information regarding the prevalence of malocclusion in primary dentition in mainland China. Furthermore, the characteristic features of malocclusion in children remain unclear. Hence, we performed this meta-analysis to systematically review the results of the initial published literature on malocclusion in Chinese pre-schoolers to investigate the prevalence of malocclusion according to malocclusion subtypes, terminal plane types, temporal trends, and gender and regional differences among Chinese children. (The study did not include data from Hong Kong, Taiwan, or Macao, because the cultural activities and socioeconomic status of individuals in these regions differ significantly from those in mainland China.) The current evaluation aimed to increase awareness among policy-makers and clinicians of the epidemiological characteristics and clinical features of malocclusion, thereby laying the groundwork for the effective prevention and timely treatment of malocclusion in primary dentition.

## Results

### Literature search and quality assessment

**Figure 1:**
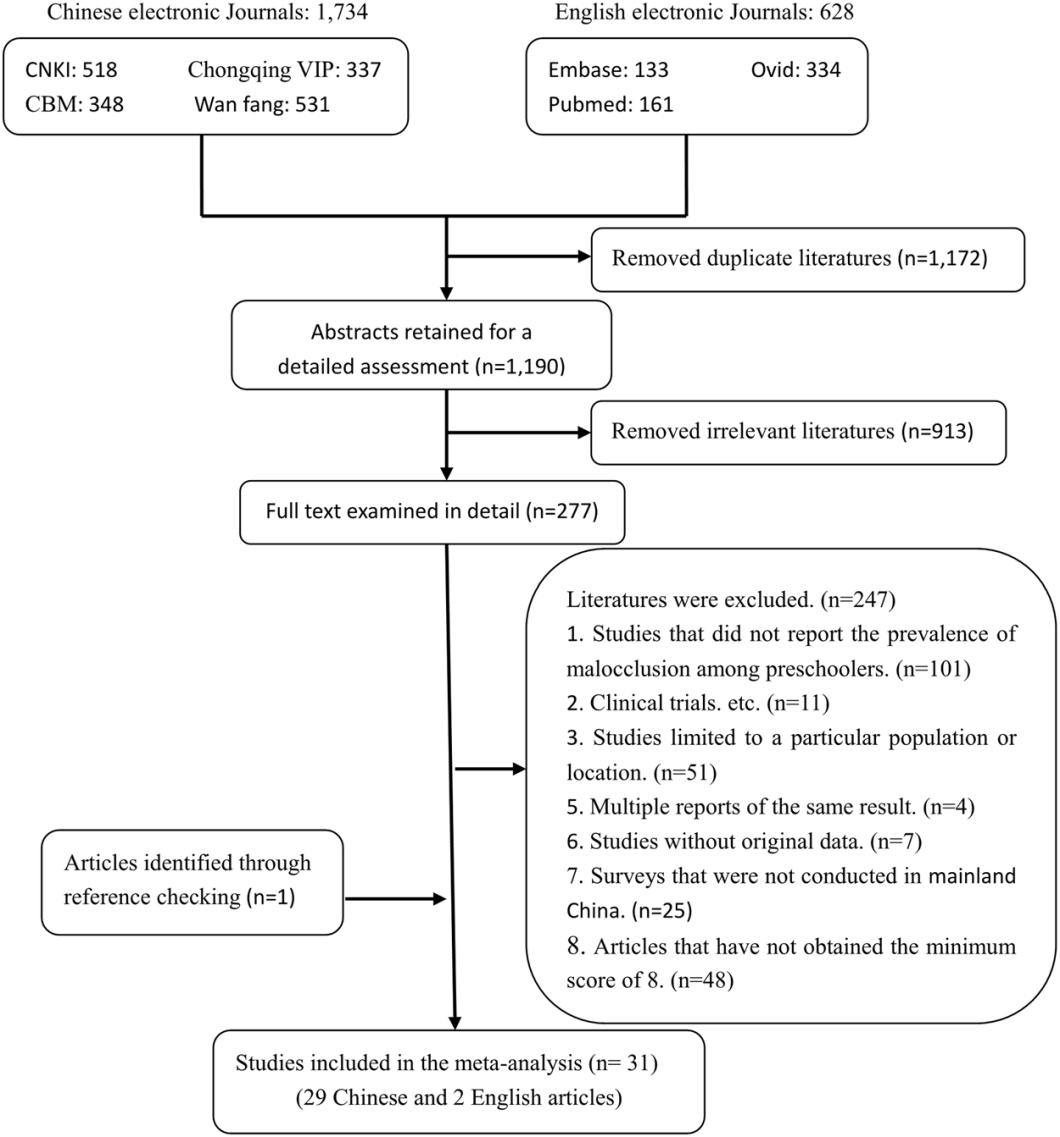
Literature search and review flowchart.

The literature search and quality assessment were performed by the first two authors (L.S. and F.H.), with an acceptable inter-examiner agreement (both kappa values > 0.782, Supplementary Table S1). A total of 31 eligible articles (29 in Chinese and 2 in English) were included in this meta-analysis. One national-level, 4 provincial-level and 26 city-level papers were included. The combined total sample was 51,100 individuals aged 2 to 7 years old. Notably, the Angle classification was used as the diagnostic criterion for malocclusion in the 29 Chinese papers, whereas the 2 English articles^12,14^ adopted a morphological classification of malocclusion from a previous study^15^. Quality assessment revealed that the general quality of the included studies was medium, and the numbers of studies scored from 8 to 12 were 5, 11, 4, 3 and 8, respectively. Furthermore, the quality of the included epidemiological studies improved over time (Supplementary Table S2). The specific search process is summarized in Fig. 1, and the basic characteristics of the 31 articles are summarized in Table 1 and Supplementary Table S3.

**Table 1:** Distribution and characteristics of the primary studies included in the meta-analysis.

First author and publication year	Language	Location of study	U/R	Score	Total sample size					Total case size							
					Male	Female	Urban	Rural	Total	Male	Female	Urban	Rural	Class I	Class II	Class III	Total
Huang Caiping 2013	C	Yu yao	U	10	164	150	—	—	314	78	62	—	—	102	17	21	140
Wang Jing 2007	C	Tang shan	U + R	9	250	192	—	—	442	122	92	—	—	119	44	51	214
Yang Hongzhen 2010	C	Bao ding	U + R	10	—	—	459	470	929	—	—	212	224	132	45	259	436
Xiao Yan 2011	C	Chang chun	U	9	1,470	1,254	—	—	2,724	—	—	—	—	—	—	—	726
Huang Ning 2005	C	Shuang liu	R	9	696	583	—	—	1,279	—	—	—	—	—	—	—	491
Yin Yanchun 2014	C	Da qing	U	11	—	-	—	—	1,904	—	—	—	—	674	149	203	1,026
Li Zhaohui 2009	C	De yang	U	10	1,709	1,504	—	—	3,213	461	295	—	—	—	—	—	756
Lv Yulin 1988	C	Fu zhou	U	8	1,610	1,492	—	—	3,102	674	681	—	—	—	—	—	1,355
Zheng Zhijun 2006	C	Gui yang	U	9	255	201	—	—	456	54	59	—	—	—	—	—	113
He Hongxu 2011	C	Ha erbing	U	10	—	—	—	—	3,956	—	—	—	—	1,637	244	279	2,160
Liu Yingqi 2009	C	Han dan	U	9	735	627	—	—	1,362	—	—	—	—	—	—	—	514
Yang Zaibo 2010	C	En shi	U + R	9	956	906	966	896	1,862	518	478	523	473	—	—	—	996
Wan Jianying 2013	C	De an	R	12	800	800	—	—	1,600	265	303	—	—	—	—	—	568
Huang Guiyue 2015	C	Kun ming	U	12	1,124	992	—	—	2,116	770	746	—	—	—	—	—	1,516
Li Haifeng 2013	C	Da lian	U	9	—	—	—	—	2,448	—	—	—	—	—	—	—	527
Qu Ling 2001	C	San ya	R	9	563	439	—	—	1,002	—	—	—	—	—	—	—	207
Yang Tao 2013	C	Chang chun	U	8	145	170	—	—	315	67	82	—	—	—	—	—	149
Sun Xinhua 1990	C	Chang chun	U	9	548	452	—	—	1,000	304	259	—	—	288	52	223	563
Feng Jinqiu 2015	C	Shang hai	U + R	12	1,421	1,323	—	—	2,744	1,041	870	—	—	—	—	—	1,911
Weng Sien 2006	C	Shang hai	U	12	239	220	—	—	459	—	—	—	—	70	70	57	197
Zhao Fengmei 1999	C	Shang hai	U	9	1,062	1,062	—	—	2,124	216	228	—	—	—	—	—	444
Zhou Xinhua 2017	E	Shang hai	U	12	1,247	1,088	—	—	2,335	1,048	912	—	—	—	—	—	1,960
Zhang Cuicui 2014	C	Shao yang	U + R	9	950	850	—	—	1,800	—	—	—	—	570	266	147	983
Chen Min 2016	C	Shao xing	U	12	292	276	—	—	568	196	201	—	—	—	—	—	397
Zhao Zhenjin 2002	C	Shen yang	U	8	—	—	—	—	591	—	—	—	—	43	22	46	111
Li Lin 1992	C	Urumchi	U	11	—	—	—	—	491	129	103	—	—	157	28	47	232
Zhou Zhifei 2016	E	Xi an	U + R	12	1,185	1,050	1,518	717	2,235	776	706	935	547	—	—	—	1,482
Liu Yuan 2015	C	Yi ning	U + R	12	517	448	596	369	965	327	279	402	204	—	—	—	606
Wang Bing 2000	C	Yin chuan	U	8	749	597	—	—	1,346	96	81	—	—	—	—	—	177
Liang Xueping 1995	C	Yun chen	U	8	—	—	—	—	109	—	—	—	—	—	—	—	63
Fu Minkui 2002	C	China	U + R	11	—	—	—	—	5,309	—	—	—	—	1,423	536	793	2,752

C: Chinese; E: English; U: Urban; R: Rural; Score: the quality assessment score.

### Prevalence by Angle classification

Approximately 23,772 children exhibited malocclusion, with a pooled prevalence of 45.50% (95% confidence interval (CI): 38.08–52.81%). Eleven articles described the prevalence of Class I, Class II and Class III malocclusion. Based on the combined results, the highest prevalence of malocclusion in mainland China pre-schoolers was Class I malocclusion at 26.50% (CI: 19.96–33.12%) compared with Class II malocclusion at 7.97% (CI: 6.06–9.87%) and Class III malocclusion at 12.60% (CI: 9.45–15.68%, Table 2).

**Table 2:** Pooled prevalence of malocclusion in primary dentition in mainland China.

Variables	Number of study	Sample size	Case size	Pooled prevalence (%)	95% CI (%)	Heterogeneity	
						Q	I^2^ (%)
Angle classification							
Malocclusion	31	51,100	23,772	45.50	38.08–52.81	10,277.82	99.7 (99.7–99.7)
Class I malocclusion	11	17,195	5,215	26.50	19.96–33.12	937.03	98.9 (98.7–99.2)
Class II malocclusion	11	17,195	1,473	7.97	6.06–9.87	205.75	95.1 (92.9–96.7)
Class III malocclusion	11	17,195	2,126	12.60	9.45–15.68	402.9	97.5 (96.6–98.2)
Types of malocclusion							
Deep overbite	17	16,203	6,336	33.66	27.66–39.67	1,020.51	98.4 (98.1–98.7)
Spacing	4	4,656	1,291	28.34	20.82–35.87	46.07	93.5 (86.5–96.9)
Anterior crossbite	18	16,730	2,778	25.29	20.01–30.58	1,872.34	99.1 (98.9–99.2)
Individual malocclusion	6	1,660	323	13.88	4.93–22.83	164.37	97.0 (95.2–98.1)
Early loss of							
primary teeth	6	7,629	516	10.46	6.72–14.19	540.17	99.1 (98.7–99.3)
Deep overjet	13	13,790	2,429	10.16	4.19–16.12	3,039.96	99.6 (99.5–99.7)
Hypodontia	4	4,234	868	8.68	3.17–14.19	61.45	95.1 (90.5–97.5)
Crowding	15	13,861	1,417	8.03	4.93–11.13	781.65	98.2 (97.7–98.6)
Anterior edge-to-edge	14	12,428	642	7.84	5.82–9.87	404.76	96.8 (95.7–97.6)
Openbite	12	14,402	495	3.36	2.24–4.48	428.66	97.4 (96.6–98.1)
Posterior crossbite	7	8,829	255	2.81	1.8–4.53	204.41	97.1 (95.6–98.1)
Posterior scissor bite	6	3,471	99	2.31	1.02–3.60	89.26	94.4 (90.3–96.8)
Terminal plane relationship of the second primary molars							
Flush terminal	8	14,562	6,855	47.10	28.76–65.44	4,617.24	99.8 (99.8–99.9)
Mesial step	8	14,562	6,341	43.24	24.85–61.63	4,506.42	99.8 (99.8–99.9)
Distal step	8	14,562	736	5.04	3.29–6.79	193.09	96.4 (94.6–97.6)
Bilateral symmetry	7	12,446	630	5.03	3.06–6.99	175.35	96.6 (94.7–97.8)
Publication year							
≤1999	5	6,826	2,657	44.98	30.35–59.60	579.87	99.3 (99.1–99.5)
2000–2004	4	8,248	3,247	26.12	4.33–47.91	1,387.28	99.8 (99.7–99.8)
2005–2009	6	7,211	2,285	35.86	27.73–43.99	244.50	98.0 (97.0–98.6)
2010–2014	10	17,852	7,711	43.89	34.56–53.21	1,550.37	99.4 (99.3–99.5)
≥2015	6	10,963	7,872	70.75	64.07–77.43	313.88	98.4 (97.7–98.7)
Gender							
Male	17	14,034	7,013	48.84	37.01–60.67	4,097.82	99.6 (99.6–99.7)
Female	17	12,703	6,334	49.55	37.57–61.53	3,821.77	99.6 (99.5–99.6)
Urban/rural area							
Urban	20	12,891	29,488	43.01	32.79–53.23	8,025.05	99.8 (99.7–99.8)
Rural	3	1,501	5,326	31.51	20.86–42.16	108.69	98.2 (96.6–99.0)

### Prevalence by malocclusion type

The meta-analysis of malocclusion types based on 14 primary articles revealed the following proportions: deep overbite 33.66% (CI: 27.66–39.67%), spacing 28.34% (CI: 20.82–35.87%), anterior crossbite 25.29% (CI: 20.01–30.58%), individual malocclusion 13.88% (CI: 4.93–22.83%), early loss of primary teeth 10.46% (CI: 6.72–14.19%), deep overjet 10.16% (CI: 4.19–16.12%), hypodontia 8.68% (CI: 3.17–14.19%), crowding 8.03% (CI: 4.93–11.13%), anterior edge-to-edge 7.84% (CI: 5.82–9.87%), openbite 3.36% (CI: 2.24–4.48%), posterior crossbite 2.81% (CI: 1.8–4.53%), and posterior scissor bite 2.31% (CI: 1.02–3.60%, Table 2).

### Prevalence by terminal plane relationship of the second primary molars

Eight articles reported the terminal plane relationship of the second primary molars in mainland China. The pooled results revealed that the most common terminal plane relationship from ages 2 to 7 was the flush terminal plane at 47.10% (CI: 28.76–65.44%), followed by the mesial step at 43.24% (CI: 24.85–61.63%), distal step at 5.04% (CI: 3.29–6.79%), and bilateral symmetry at 5.03% (CI: 3.06–6.99%, Table 2).

### Prevalence over time

**Figure 2:**
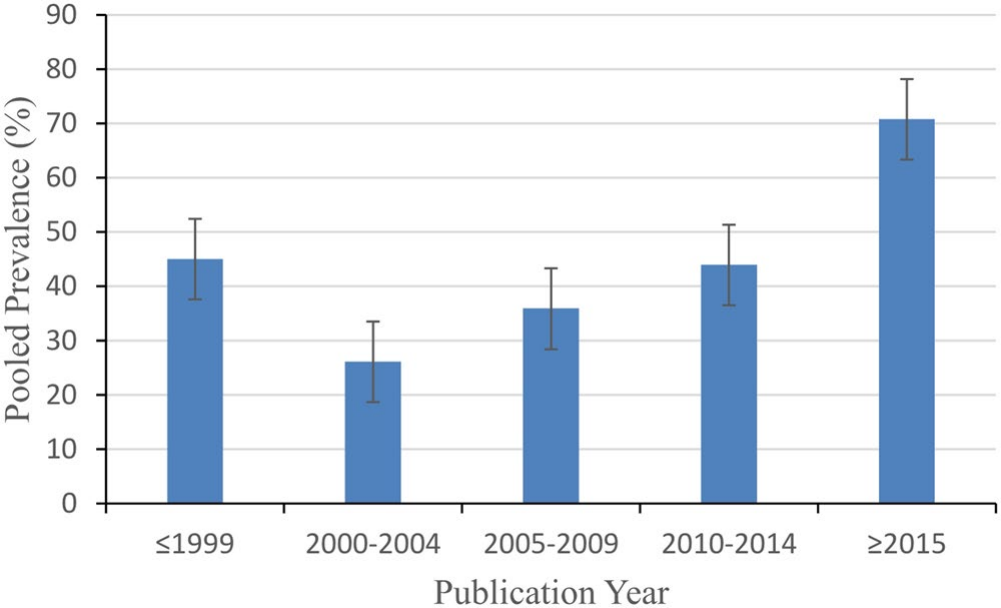
Temporal trend of malocclusion prevalence in primary dentition in mainland China from 1988 to 2017.

The year of study publication varied from 1988 to 2017. Following the methods of previous reviews^16,17^, we divided the articles into the following 5 periods: ≤1999, 2000–2004, 2005–2009, 2010–2014 and ≥2015. The overall prevalence of malocclusion in children during these periods were 44.98% (CI: 30.35–59.60%), 26.12% (CI: 4.33–47.91%), 35.86% (CI: 27.73–43.99%), 43.89% (CI: 34.56–53.21%) and 70.75% (CI: 64.07–77.43%), respectively (Table 2). This finding clearly demonstrates a substantial increasing trend in deciduous-dentition malocclusion over time (Fig. 2).

### Prevalence by geographic region

**Figure 3:**
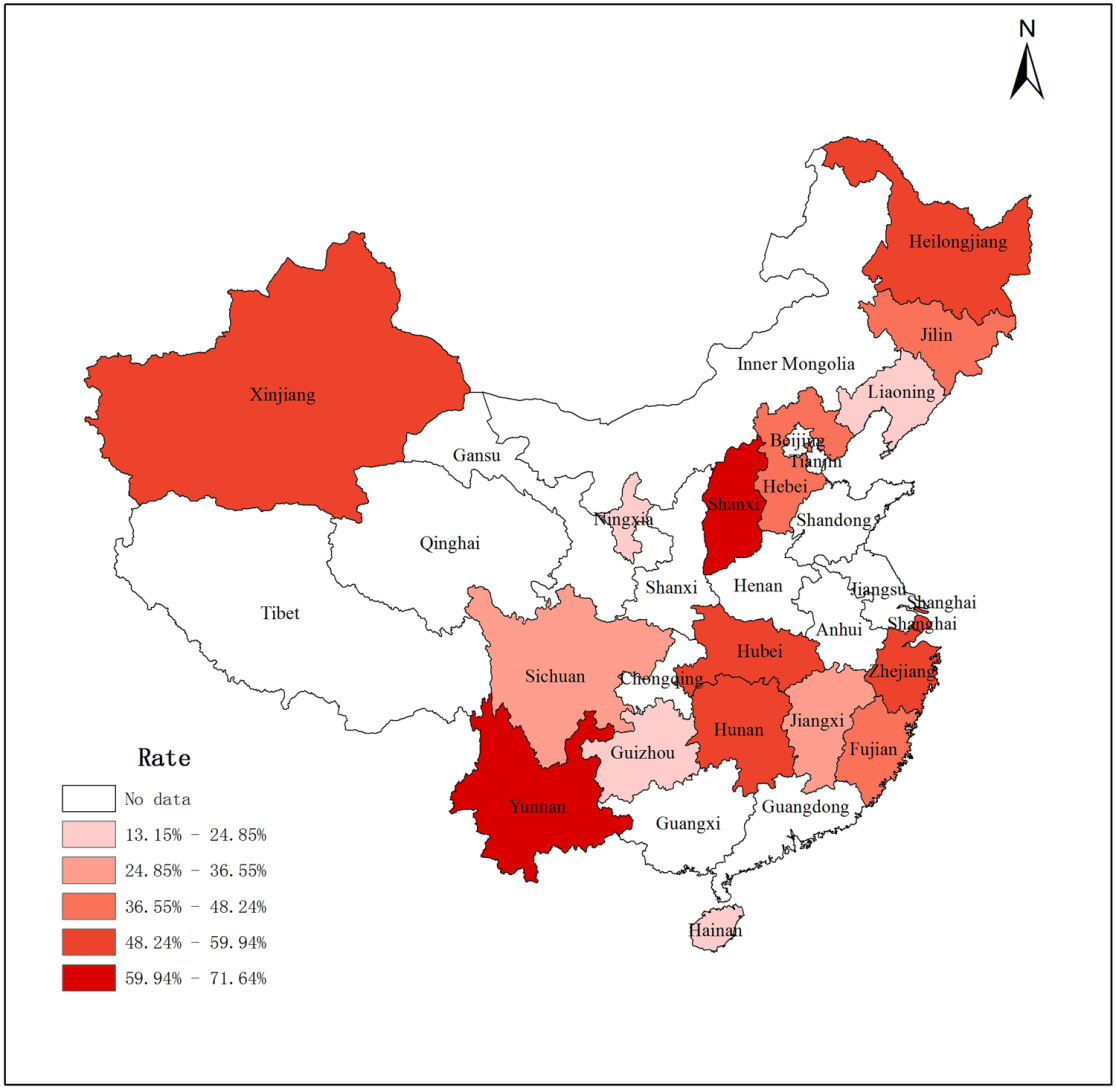
Spatial distribution of the prevalence of malocclusion in mainland China pre-schoolers (created by the ArcGIS 10.0).

The available data from 30 articles was combined, encompassing 14 provinces, 2 autonomous regions, and 1 municipality in mainland China. There were statically significant variations in the prevalence of malocclusion across provinces (Supplementary Table S4). The highest prevalence rates (indicated by the darkest red on the map in Fig. 3) were in Zhejiang at 57.32% (CI: 32.52–82.12%), Yunnan at 71.64% (CI: 69.72–73.57%) and Shanxi at 63.31% (CI: 55.34–71.28%). The areas of lowest prevalence appeared to be in Ningxia at 13.15% (CI: 11.34–14.96%), Liaoning at 20.50% (CI: 17.89–23.10%) and Hainan at 20.66% (CI: 18.15–23.17%), as indicated by light red on the map in Fig. 3.

### Prevalence by gender

**Figure 4:**
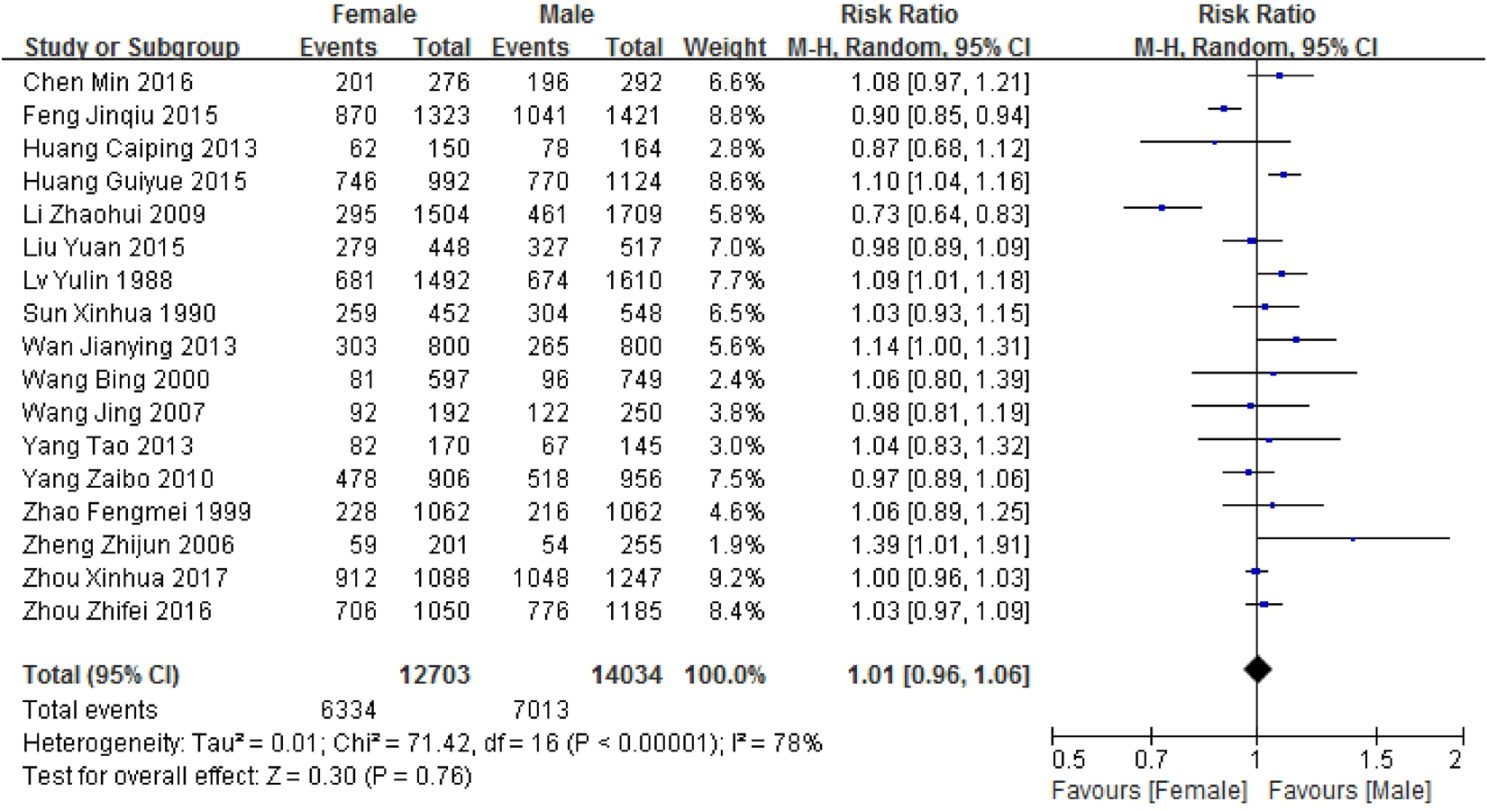
Forest plot of the prevalence of malocclusion in primary dentition among different genders in mainland China.

Seventeen articles reported the prevalence of malocclusion among 14,034 males and 12,703 females aged 2–7 years in mainland China. The prevalence of malocclusion in primary dentition in males and females was 48.84% (CI: 37.01–60.67%) and 49.55% (CI: 37.57–61.53%), respectively (Table 2). Figure 4 demonstrates that there was no significant difference (relative risk (RR) = 1.01, [0.96–1.06]) in malocclusion prevalence by gender.

### Prevalence by urban/rural area

**Figure 5:**
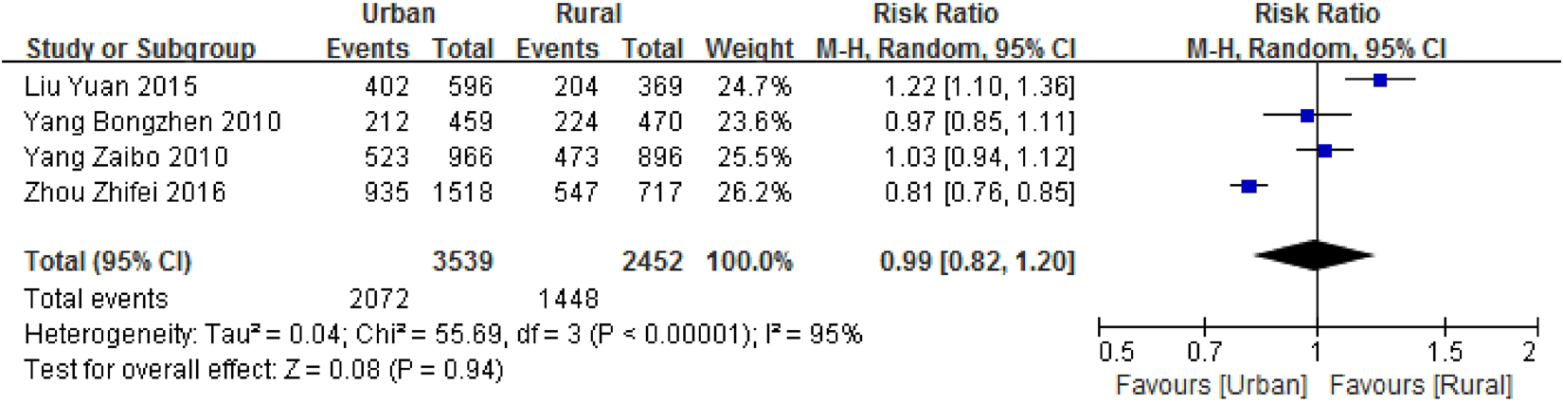
Forest plot of the prevalence of malocclusion in primary dentition among rural and urban areas of mainland China.

Only 4 eligible articles were combined to evaluate the pooled prevalence of malocclusion in pre-schoolers in urban and rural areas. The pooled prevalence of malocclusion was 43.01% (CI: 32.79–53.23%) for urban children and 31.51% (CI: 20.86–42.16%) for rural pre-schoolers (Table 2). No significant difference was found between rural and urban areas (RR = 0.99, [0.82–1.20], Fig. 5).

### Heterogeneity and publication bias

**Figure 6:**
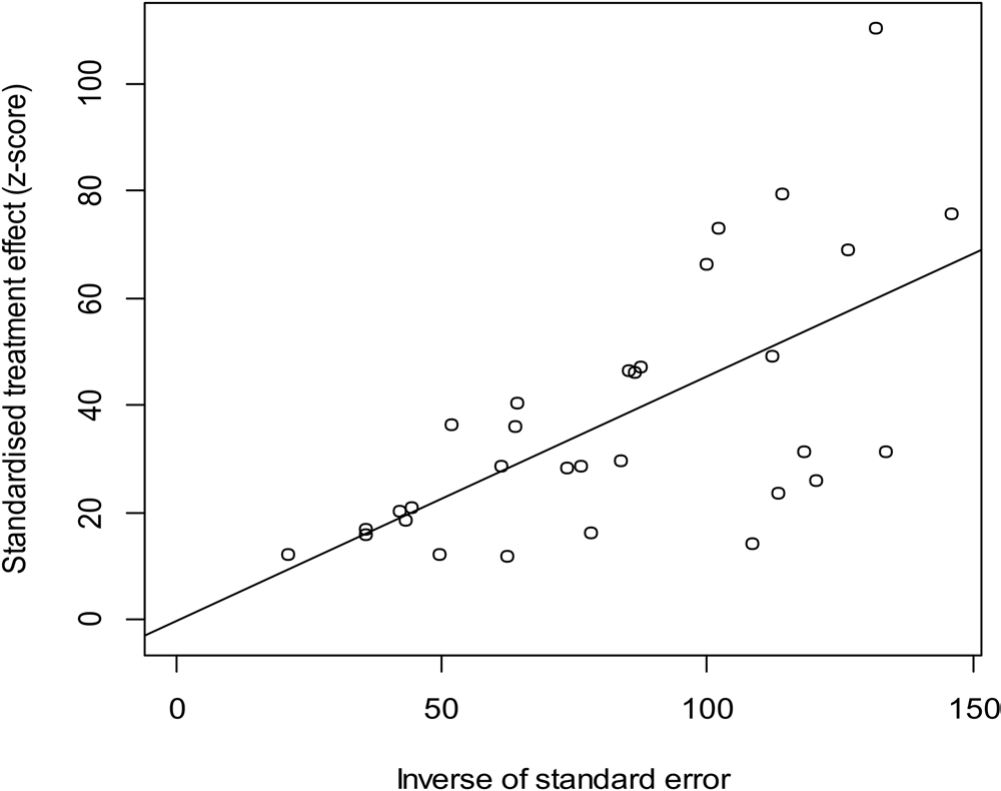
Egger’s test for publication bias.

To identify the source of high heterogeneity, susceptible variables, including a high prevalence of malocclusion, were entered in a meta-regression model. Publication year (p = 0.043), diagnostic method (p = 0.036), and urban/rural area (p < 0.0001), rather than geographical distribution (in southern China or northern China) (p = 0.6168, Supplementary Table S5), largely explained the estimated heterogeneity. Furthermore, Egger’s test for publication bias was skewed, and the values, which were approximately evenly distributed around the overall mean estimate, suggested insignificant publication bias (Fig. 6).

## Discussion

This meta-analysis combined the results of initial studies, providing a more accurate and comprehensive picture of the prevalence of deciduous-dentition malocclusion in mainland China based on a random effects model. The results indicated that almost 45.5% of children in mainland China suffer from at least one type of malocclusion. Moreover, a high level of variation was observed among different provinces, which may be due to differences in criteria, ethnic groups, age groups, registration procedures or environmental and genetic factors^18,19^.

Compared to the two other Angle classifications, the highest estimated prevalence of Class I malocclusion was 26.5%. To better understand this result, it is helpful to consider a systematic review of malocclusion prevalence among Iranian children, which concluded that the excessive consumption of sugars, which causes caries and the early loss of deciduous teeth, as well as a lack of hygiene or healthcare, increased the prevalence of Class I malocclusion^20^. The rate of Class III malocclusion in children was 12.6%. In addition to genetic factors, the habit of mandibular protrusion and incorrect feeding habits, such as nursing while lying down, increased the incidence of Class III malocclusion^21^. The lowest prevalence rate of 7.97% was observed in Class II malocclusion. However, early recognition of such discrepancies is required, as a previous longitudinal study suggested that Class II malocclusion in the deciduous dentition might determine a related Class II permanent-dentition malocclusion^22^.

The most common trait of malocclusion was revealed to be a deep overbite (33.66%), which is consistent with a previous study^23^. However, deepbite in primary dentition may be temporary, and spontaneous correction may occur due to vertical growth of the mandibular ramus and the full eruption of permanent molars^24^, as a longitudinal study supported^25^. Scholars have recommended that an increased overbite in primary dentition is rarely treated unless the lower incisors impinge the maxillary palatal mucosa, leading to pain or other clinical symptoms^26^. Physiological spaces, including the developmental spaces and the primate space, were the second most common characteristic in primary dentition, with a prevalence of 27%. Spacing indicates proper alignment of the permanent dentition^27,28^; thus, early effective treatment is not necessary in such cases. Crossbite (25.29%), mainly associated with genetically inherited characteristics and environmental factors, was observed at a higher prevalence than that reported in India^29^. Self-correction was noted for anterior crossbite^8^, whereas posterior crossbite was transferred from the deciduous to the permanent dentition^29^. Likewise, crossbite warrants increased early check-ups and treatment to decrease the long-term effects on growth and development. Relative to the reported prevalence of increased overjet in the primary dentition in different countries, which ranges from 3% to 16%^30,31^, the prevalence rate in the present study was moderate, at 10.16%. Because increased overjet was alleviated during the transition from primary to permanent dentition, it was not a precise indicator of a similar increase in the permanent dentition. In addition, a standard of 3 mm is acceptable to clinicians. The prevalence of crowding (8.03%) was much lower than that reported in Colombia (52.1%)^32^. This difference might be the result of a sampling error secondary to the small sample size in the former study. There are clear indications that crowding in deciduous dentition, mostly due to the modern diet, contributes to the disproportion between the jaw and tooth size in the permanent stage^30^; thus, early interceptive treatment is necessary to enhance the favorable growth and development of the jaw and teeth^33,34^. Moreover, the discrimination between temporary malocclusion and pathological malocclusion is crucial in this period.

The present study demonstrated that the majority of children in mainland China had a flush terminal plane (47.10%), followed by a mesial step relationship (43.24%) and a distal step relationship (5.04%). In India, a survey of 1,000 pre-schoolers revealed similar results; the molar relationship prevalence was the flush terminal plane (66.0%), mesial step (12.8%), distal step (2.4%) and bilateral symmetry (18.8%)^29^. There was a consensus that the distal surfaces on the second deciduous molars, i.e., the primary molar relationship in the deciduous dentition, predicted the identification of the permanent molar relationship^35^. A previous survey^7^ revealed that most cases of flush and mesial terminal plane developed into Angle Class I in the permanent dentition due to a combination of forward movement of the mandible and mesial migration of the mandibular arcus^36^. Additionally, a study by Ravn^37^ demonstrated that a distal step molar relationship could develop into an Angle Class II molar relationship in permanent dentition. Therefore, additional longitudinal studies are necessary to identify the changes in the molar relationship from primary dentition to permanent dentition.

Our study revealed a dramatic increasing trend in the prevalence of malocclusion over time among children in mainland China, which suggests the need for the early recognition and treatment of deciduous-dentition malocclusions in further diagnostic and treatment procedures. There are several potential reasons for this observation. First, the rapid development of China’s economy has changed lifestyles and diet structures among its inhabitants, and the overconsumption of refined and high-sugar foods has increased the risk of malocclusion^38,39^. Second, a lack of proper oral hygiene knowledge, attitudes or behaviors among parents has likely contributed to the increasing prevalence of malocclusion among children^40^. For example, there is a general misunderstanding that malocclusion in primary dentition is temporary and should resolve with the eruption of permanent teeth. Additionally, the government’s investment in oral health services and resources^41^, as well as health education programmes^42^, is insufficient, especially in rural areas.

No pattern was observed in the geographical distribution of deciduous-dentition malocclusion in mainland China. For example, similar prevalence rates were observed in scattered locations such as Yunnan and Shanxi. The wide variation in malocclusion prevalence among different regions might partly be due to regional differences in socioeconomic status, eating habits and cultural environments^43^. The prevalence map in Fig. 3 can be used as a reference for oral health services and malocclusion resources, although it may not be fully representative of Chinese pre-schoolers due to insufficient information.

There has been a fervent debate about the prevalence of malocclusion in primary dentition between genders. Previous studies^43–45^ reported that girls had a higher risk of malocclusion than boys; however, Li Zhaohui^46^ and Girish R Shavi *et al*.^28^ identified a significantly higher malocclusion prevalence among boys. Our pooled results demonstrated no significant gender difference (RR = 1.01, CI: 0.88–1.17), which reinforced the results of the majority of other relevant studies^11,12,47^. Consequently, we inferred that gender likely has no significant effect on malocclusion in children. However, further gender-based cohort studies are warranted to confirm this assumption.

Our study found that rural children had a malocclusion prevalence rate similar to that of their urban counterparts. This finding is inconsistent with a previous survey^23^ reporting that children with a higher socioeconomic status had a higher prevalence of malocclusion. Our finding might have been partially due to reductions in the differences between urban and rural areas caused by rapid economic development. However, we should acknowledge that the included studies covered more urban than rural areas, which might bias country-level estimates in China. Therefore, further investigations are required to more precisely define the prevalence of malocclusion in urban and rural areas.

The present study has some inherent limitations. First, statistically significant heterogeneity was observed between the primary studies. Therefore, it was impossible to identify the effects of susceptible variables on pooled prevalence. Second, all the papers included in this review were cross-sectional studies, which inevitably imposed limitations on the estimation of malocclusions due to a wide variety of tools, methods and subjective judgments. In addition, the quality of the primary studies included in this systematic review is a cause for concern. Despite the clear description of the research question and target population, most lower-quality studies failed to report the role of researchers, the sampling structure or the data collection method completely, which may lead to biased and inaccurate estimates of prevalence. Finally, due to the insufficient number of articles, we had to limit the study to a specific city or region as a representative sample, which may or may not precisely represent the population.

This systematic review identified 31 published studies that estimated the prevalence of malocclusion among mainland China pre-schoolers. Our findings reveal that deciduous-dentition malocclusion has become a serious and urgent problem. Based on the mentioned points, we hope that these findings will be considered by epidemiologists and clinicians. Furthermore, the increasing trend in malocclusion highlights the need for policy makers to invest in efforts to improve oral health and the early preventive treatment of deciduous-dentition malocclusion in mainland China.

## Methods

This systematic review was performed according to the Preferred Reporting Items of Systematic reviews and Meta-Analyses (PRISMA) guidelines^48^ (Supplementary Checklist S1). Prior to the study, a training session was conducted to clarify the procedure of this systematic review, creating correct methods for full text examination and quality assessment. Kappa coefficients^49^ were calculated to assess inter-examiner variability.

### Search strategy

A pilot search was conducted by a research team with experts in orthodontics, epidemiology and statistics. Subsequently, a comprehensive literature search strategy was applied using the following keywords: ‘prevalence’, ‘frequency’, ‘epidemiological’, ‘dental malocclusion’, ‘Class I’, ‘Class II’, ‘Class III’, ‘preschool children’, ‘child’, and ‘China’ (until March 2017). The search was performed in the following seven Chinese and English databases: Chinese Biomedical Literature Database (CBM) (1978-), Chinese National Knowledge Infrastructure database (CNKI) (1979-), Chinese Wan Fang database (1990-), Chongqing VIP database (1989-), PubMed (1966-), EMBASE (1974-), and Ovid (1984-). The final search evaluation was performed by the first author (L.S.), and the search results were merged using bibliographic citation management software EndNote X7 (Thomson Reuters, CA, USA). Meanwhile, a manual search was applied to the reference lists of all the eligible articles for potentially relevant studies.

### Selection criteria

Titles and abstracts were screened independently by two authors (L.S. and F.H.), and duplicate and irrelevant records were removed. The inter-examiner variability statistic was acceptable (kappa value = 0.782, Supplementary Table S1a). The full texts of potentially eligible studies were examined. We selected original English/Chinese surveys that obtained the required quality scores and reported the minimum information (number of subjects and number of malocclusion events) necessary to calculate the pooled prevalence of malocclusion in primary dentition based on random sampling. Studies with the following features were excluded: (1) studies that did not report the prevalence of malocclusion among pre-schoolers; (2) clinical trials, abstracts, conference proceedings, commentaries, case–control studies or case report studies; (3) studies in which the participants were limited to a particular occupation, population, community group or location; (4) studies that did not report the most detailed data for which other articles based on the same sample were available; (5) studies that did not provide minimum information to calculate prevalence; (6) surveys that were not conducted in mainland China; and (7) articles that did not obtain a quality minimum score of 8. The inter-examiner variability for this stage was substantial (kappa value = 0.795, Supplementary Table S1b), and disagreements were resolved by discussion or by the study’s primary designer (J.H.W.).

### Quality assessment

We used a checklist based on the guidelines of the Strengthening the Reporting of Observational Studies in Epidemiology (STROBE) to assess the quality of the selected studies^50^. The checklist contains 12 questions that aim to assess the purpose of the survey, the appropriateness of the research design, sample size, sampling methods, population, data collection tools, variable evaluation status, statistical analyses, and appropriate means of reporting claims based on objectives^51^ (Supplementary Checklist S2). The options for each question are ‘yes = 1 point’ or ‘no = 0 points’; thus, a maximum score of 12 points represents the highest quality. The assessment was performed by two trained authors (L.S. and F.H.) with a high inter-examiner agreement (kappa value = 0.795, Supplementary Table S1b), and disputes were settled by consensus or by the study’s primary designer (J.H.W.) when necessary. Finally, surveys that received a score of 8 or higher were selected for the meta-analysis.

### Data extraction

The following variables were extracted by the first two authors (L.S. and F. H.) independently: (1) publication details, including article title, first author, year of publication and language of the article; (2) research design, including the study location and period, sampling methods, diagnostic criteria, characteristics of the participants and total sample size; (3) details of the target indicators, including the total case size and overall prevalence in different genders, region types (i.e., urban or rural), Angle classifications (Classes I, II, III and normal), malocclusion types, and the terminal plane relationships of the second primary molars. We contacted the corresponding authors for further or missing information as needed and resolved disagreements by consensus or the decision of the third author (J.H.W.) if necessary. The final data were entered into an Excel spreadsheet for analysis.

### Statistical analysis

A meta-analysis was conducted using STATA software version 12.0 (Stata, College Station, TX, USA). The standard error of each indicator in all eligible studies was calculated based on the binomial distribution formula. Subsequently, tests of heterogeneity among the studies were evaluated using Q and I^2^ tests. According to the heterogeneity results, a random effects model (I^2^ > 50% or p < 0.05) or a fixed effects model (I^2 < ^50% or p > 0.05) was used to estimate the pooled prevalence of Class I, II, and III malocclusion; malocclusion types; and the terminal plane relationship of the second primary molars. A subgroup analysis was conducted to identify the source of high heterogeneity, and possible factors (gender and location of residence) were examined by risk ratios (RR) and 95% confidence intervals (CI) in the Cochrane Review Manager Version 5.1. To evaluate the temporal trend in malocclusion in pre-schoolers, available studies were categorized into five data collection time periods as follows: ≤1999, 2000–2004, 2005–2009, 2010–2014, and ≥2015. In addition, the pooled prevalence estimated for deciduous-dentition malocclusion in each province was entered into ArcGIS Version 10 software to map the geographical distribution.

## Electronic supplementary material

supplementary information
