# Supplementary material for: Prevalence of malocclusion in primary dentition in mainland China, 1988–2017: a systematic review and meta-analysis
Source: Sci Rep. 2018 Mar 16;8:4716. doi: 10.1038/s41598-018-22900-x (PMC5856803; doi:10.1038/s41598-018-22900-x)
Supplement: Supplementary file 1 — supplementary information [file 41598_2018_22900_MOESM1_ESM.pdf]

Prevalence of malocclusion in primary dentition in mainland China, 1988-2017: a systematic review and meta-analysis

Lu Shen[1,2,3§], Fang He[4§], Cai Zhang[1,2,3], Haofeng Jiang[1,2,3], Jinhua Wang[1,2,3*]

[1]College of Stomatology, Chongqing Medical University, Chongqing, 401147, China.

[2]Chongqing Key Laboratory of Oral Diseases and Biomedical Sciences, Chongqing, 401147, China.

[3]Chongqing Municipal Key Laboratory of Oral Biomedical Engineering of Higher Education, Chongqing, 401147, China.

[4]School of Public Health and Management, Chongqing Medical University, Research Center for Medicine and Social Development, Collaborative Innovation Center of Social Risks Governance in Health, Chongqing Medical University, Chongqing, 400016, China.

[§]First co-authors

[*]Corresponding author

Correspondence to:

Jinhua Wang, College of Stomatology, Chongqing Medical University, Chongqing, China, No.426 Songshibei Road, Yubei District, Chongqing, China; Tel: +86 23 89035824; Fax: +86 23 89035721; E-mail: dentistwjh@163.com

Table S1 | Agreement among authors [kappa coefficients]

Table S1a | Agreement among authors on titles and abstracts [kappa coefficients]

		Author 2		Total
		Inclusion	Exclusion	
Author 1	Inclusion	277	49	326
	Exclusion	55	809	864
	Total	332	858	1,190

K1=0.782

Table S1b | Agreement among authors on full-text analysis [kappa coefficients]

		Author 2		Total
		Inclusion	Exclusion	
Author 1	Inclusion	30	8	38
	Exclusion	5	234	239
	Total	35	242	277

K2=0.795

Table S2 | Quality assessment of the 31 included studies

First author & publication year	Questions												Score
	1	2	3	4	5	6	7	8	9	10	11	12	
Huang Caiping 2013	Y	Y	Y	Y	N	N	Y	Y	Y	Y	Y	Y	10
Wang Jing 2007	Y	Y	Y	N	Y	N	N	Y	Y	Y	Y	Y	9
Yang Hongzhen 2010	Y	Y	Y	Y	N	Y	N	Y	Y	Y	Y	Y	10
Xiao Yan 2011	Y	Y	Y	Y	Y	Y	Y	N	N	Y	N	Y	9
Huang Ning 2005	Y	Y	Y	Y	Y	N	Y	N	N	Y	Y	Y	9
Yin Yanchun 2014	Y	Y	Y	Y	Y	Y	Y	Y	N	Y	Y	Y	11
Li Zhaohui 2009	Y	Y	Y	Y	Y	Y	Y	N	N	Y	Y	Y	10
Lv Yulin 1988	Y	Y	Y	N	Y	N	Y	N	N	Y	Y	Y	8
Zheng Zhijun 2006	Y	Y	Y	Y	Y	N	Y	N	N	Y	Y	Y	9
He Hongxu 2011	Y	Y	Y	Y	Y	Y	Y	Y	N	Y	N	Y	10
Liu Yingqi 2009	Y	Y	Y	Y	Y	N	Y	N	N	Y	Y	Y	9
Yang Zaibo 2010	Y	Y	Y	Y	Y	N	Y	N	N	Y	Y	Y	9
Wan Jianying 2013	Y	Y	Y	Y	Y	Y	Y	Y	Y	Y	Y	Y	12
Huang Guiyue 2015	Y	Y	Y	Y	Y	Y	Y	Y	Y	Y	Y	Y	12
Li Haifeng 2013	Y	Y	Y	Y	Y	N	Y	N	Y	Y	N	Y	9
Qu Ling 2001	Y	Y	Y	Y	Y	N	Y	N	Y	Y	N	Y	9
Yang Tao 2013	Y	Y	Y	N	Y	N	N	N	Y	Y	Y	Y	8
Sun Xinhua 1990	Y	Y	Y	Y	Y	N	Y	N	N	Y	Y	Y	9
Feng Jinqiu 2015	Y	Y	Y	Y	Y	Y	Y	Y	Y	Y	Y	Y	12
Weng Sien 2006	Y	Y	Y	Y	Y	Y	Y	Y	Y	Y	Y	Y	12
Zhao Fengmei 1999	Y	Y	Y	Y	Y	N	Y	N	N	Y	Y	Y	9
Zhou Xinhua 2017	Y	Y	Y	Y	Y	Y	Y	Y	Y	Y	Y	Y	12
Zhang Cuicui 2014	Y	Y	Y	Y	Y	Y	N	N	N	Y	Y	Y	9
Chen Min 2016	Y	Y	Y	Y	Y	Y	Y	Y	Y	Y	Y	Y	12
Zhao Zhenjin 2002	Y	Y	Y	Y	N	N	Y	N	N	Y	Y	Y	8
Li Lin 1992	Y	Y	Y	Y	Y	Y	Y	Y	N	Y	Y	Y	11
Zhou Zhifei 2016	Y	Y	Y	Y	Y	Y	Y	Y	Y	Y	Y	Y	12
Liu Yuan 2015	Y	Y	Y	Y	Y	Y	Y	Y	Y	Y	Y	Y	12
Wang Bing 2000	Y	Y	Y	N	Y	N	Y	N	N	Y	Y	Y	8
Liang Xueping 1995	Y	Y	Y	N	Y	N	Y	N	N	Y	Y	Y	8
Fu Minkui 2002	Y	Y	Y	Y	Y	Y	Y	Y	N	Y	Y	Y	11

Y: Yes; N: No; Score: the quality assessment score

Table S3a | Characteristics of the 31 included studies

First author & publication year	Urban/rural areas	Crowding	Spacing	Deep overbite	Deep overjet	Anterior crossbite	Posterior crossbite	Posterior scissor bite	Anterior edge-to-edge	Openbite	Individual malocclusion	Hypodontia	Early loss of primary teeth	Total
Xiao Yan 2011	U	20	-	182	-	402	6	1	28	-	-	-	87	726
Huang Ning 2005	R	20	-	157	10	137	12	3	63	16	-	37	35	490
Liu Yingqi 2009	U	47	-	147	-	171	5	2	52	1	-	-	89	514
Yang Zaibo 2010	U+R	56	-	201	-	282	42	38	63	49	-	-	265	996
Wan Jianying 2013	R	17	-	258	49	197	-	-	38	9	-	-	-	568
Huang Guiyue 2015	U	384	-	820	2	204	-	-	84	6	-	-	16	1,516
Li Haifeng 2013	U	67	99	-	-	194	-	-	-	-	167	-	-	527
Qu Ling 2001	R	8	-	12	17	40	-	6	102	22	-	-	-	207
Yang Tao 2013	U	29	-	20	13	43	-	-	8	-	5	31	-	149
Sun Xinhua 1990	U	-	-	206	50	91	-	49	43	-	99	-	-	538
Feng Jinqiu 2015	U+R	583	-	1,520	667	330	-	-	-	198	-	65	24	3,387
Weng Sien 2006	U	16	129	137	4	41	-	-	-	0	-	-	-	327
Zhao Fengmei 1999	U	8	-	195	28	177	-	-	31	5	-	-	-	444
Zhou Xinhua 2017	U	151	1,046	1,488	791	187	14	-	54	10	-	-	-	3,741
Li Lin 1992	U	8	0	91	14	22	7	0	18	14	18	16	-	208
Zhou Zhifei 2016	U+R	-	-	840	782	152	169	-	55	156	-	-	-	2,154
Wang Bing 2000	U	-	-	43	-	90	-	-	3	9	32	-	-	177
Liang Xueping 1995	U	3	17	19	2	18	-	-	-	-	2	-	-	61

C: Chinese; E: English; U: Urban; R: Rural; Score: the quality assessment score

Table S3b | Characteristics of the 31 included studies

First author & publication year	Language	Location of study	U/R	Score	Flush terminal	Mesial step	Distal step	Bilateral symmetry	Total
Huang Caiping 2013	C	Yu yao	U	10	83	39	10	8	140
Xiao Yan 2011	C	Chang chun	U	9	1,704	804	114	102	2,724
Huang Ning 2005	C	Shuang liu	R	9	295	902	30	52	1,279
Liu Yingqi 2009	C	Han dan	U	9	852	402	57	51	1,362
Yang Zaibo 2010	C	En shi	U+R	9	1,102	565	108	87	1,862
Huang Guiyue 2015	C	Kun ming	U	12	184	1,840	92	-	2,116
Feng Jinqiu 2015	C	Shang hai	U+R	12	1,732	891	61	60	2,744
Zhou Xinhua 2017	E	Shang hai	U	12	903	898	264	270	2,335

C: Chinese; E: English; U: Urban; R: Rural; Score: the quality assessment score

Table S4 | Pooled prevalence of malocclusion in primary dentition across provinces in mainland China

Provinces	Number of study	Sample size	Cases size	Pooled prevalence (%)	95% CI (%)	Heterogeneity	
						Q	I^2 (%)
Zhe jiang	2	882	537	57.32	32.52-82.12	55.35	98.2 (95.9-99.2)
He bei	3	2,733	1,164	44.21	37.10-51.32	26.66	92.5 (81.3-97.0)
Ji lin	3	4,039	1,438	43.37	21.76-64.98	299.59	99.3 (99.0-99.6)
Si chuan	2	4,492	1,247	30.92	16.35-45.48	91.65	98.9 (97.8-99.5)
Hei longjiang	2	5,860	3,186	54.37	53.09-55.64	0.26	0.0
Fu jian	1	3,102	1,355	43.68	41.94-45.43	-	-
Gui zhou	1	456	113	24.78	20.82-28.74	-	-
Hu bei	1	1,862	996	53.49	51.23-55.76	-	-
Jiang xi	1	1,600	568	35.50	33.16-37.84	-	-
Yun nan	1	2,116	1,516	71.64	69.72-73.57	-	-
Liao ning	2	3,039	638	20.50	17.89-23.10	2.31	56.0 (0.0-89.6)
Hai nan	1	1,002	207	20.66	18.15-23.17	-	-
Shang hai	4	7,662	4,512	54.37	23.14-8.559	3,131.52	99.9 (99.9-99.9)
Hu nan	1	1,800	983	54.61	52.31-56.91	-	-
Xin jiang	2	1,456	838	55.11	39.87-70.34	32.24	96.9 (91.8-98.8)
Shan xi	2	2,344	1,545	63.31	55.34-71.28	3.10	67.7 (0.0-92.7)
Ning xia	1	1,346	177	13.15	11.34-14.96	-	-

Table S5 | Subgroup analysis of heterogeneity

Group	Number of studies	Detection rate (%)	95% CI (%)	Heterogeneity (I^2)	
				Q	I^2 (%)
**Geographic distribution**					
Southern China	12	42.2	31.49-53.67	3,845.89	99.5
Northern China	18	46.0	3.55-55.74	2,193.82	99.6
**Publication year**					
>2002	22	49.1	40.91-57.26	4,515.16	99.5
≤2002	9	34.5	24.47-46.06	1,329.03	99.4
**Diagnostic methods**					
Angle classification	29	42.4	36.25-48.71	4,724.20	99.4
Morphological classification	2	76.2	55.17-89.30	184.17	99.5
**Urban/rural area**					
Urban	20	42.0	32.95-51.67	4,503.53	99.6
Rural	3	31.0	22.04-41.56	87.92	97.7

Checklist S1. PRISMA Checklist

Section/ topic	#	Checklist item	Reported section #
**TITLE**			
Title	1	Identify the report as a systematic review, meta-analysis, or both.	Title
**ABSTRACT**			
Structured summary	2	Provide a structured summary including, as applicable: background; objectives; data sources; study eligibility criteria, participants, and interventions; study appraisal and synthesis methods; results; limitations; conclusions and implications of key findings; systematic review registration number.	Abstract
**INTRODUCTION**			
Rationale	3	Describe the rationale for the review in the context of what is already known.	Introduction - 1st to 3rd paragraphs
Objectives	4	Provide an explicit statement of questions being addressed with reference to participants, interventions, comparisons, outcomes, and study design (PICOS).	Introduction – 4th paragraph
**METHODS**			
Protocol and registration	5	Indicate if a review protocol exists, if and where it can be accessed (e.g., Web address), and, if available, provide registration information including registration number.	N/A
Eligibility criteria	6	Specify study characteristics (e.g., PICOS, length of follow-up) and report characteristics (e.g., years considered, language, publication status) used as criteria for eligibility, giving rationale.	Methods –Search strategy, Selection criteria and data extraction
Information sources	7	Describe all information sources (e.g., databases with dates of coverage, contact with study authors to identify additional studies) in the search and date last searched.	Methods – Search strategy and data extraction
Search	8	Present full electronic search strategy for at least one database, including any limits used, such that it could be repeated.	Methods – Search strategy
Study	9	State the process for selecting studies (i.e.,	Methods –Selection

selection		screening, eligibility, included in systematic review, and, if applicable, included in the meta-analysis).	criteria
Data collection process	10	Describe method of data extraction from reports (e.g., piloted forms, independently, in duplicate) and any processes for obtaining and confirming data from investigators.	Methods –Selection criteria and data extraction
Data items	11	List and define all variables for which data were sought (e.g., PICOS, funding sources) and any assumptions and simplifications made.	Methods –Data extraction
Risk of bias in individual studies	12	Describe methods used for assessing risk of bias of individual studies (including specification of whether this was done at the study or outcome level), and how this information is to be used in any data synthesis.	Methods –Quality assessment, Table S1 and Checklist S2
Summary measures	13	State the principal summary measures (e.g., risk ratio, difference in means).	Methods – Statistical analysis
Synthesis of results	14	Describe the methods of handling data and combining results of studies, if done, including measures of consistency (e.g., I^2) for each meta-analysis.	Methods – Statistical analysis
Risk of bias across studies	15	Specify any assessment of risk of bias that may affect the cumulative evidence (e.g., publication bias, selective reporting within studies).	Methods – Statistical analysis
Additional analyses	16	Describe methods of additional analyses (e.g., sensitivity or subgroup analyses, meta-regression), if done, indicating which were pre-specified.	Methods – Statistical analysis
**RESULTS**			
Study selection	17	Give numbers of studies screened, assessed for eligibility, and included in the review, with reasons for exclusions at each stage, ideally with a flow diagram.	Figure 1
Study characteristics	18	For each study, present characteristics for which data were extracted (e.g., study size, PICOS, follow-up period) and provide the citations	Results –1st paragraph, Table 1 and Table S3
Risk of bias within studies	19	Present data on risk of bias of each study and, if available, any outcome level assessment (see item 12).	Results –1st paragraph, Table S1 and Table S2
Results of individual studies	20	For all outcomes considered (benefits or harms), present, for each study: (a) simple summary data for each intervention group (b) effect estimates and confidence intervals, ideally with a forest plot.	Results –2nd to 8th paragraphs, Table1, Table S3, Figure 4 and Figure 5

Synthesis of results	21	Present the main results of the review. If meta-analyses are done, include for each, confidence intervals and measures of consistency.	Results –2nd to 8th paragraphs, Table 2, Table S4, Figure2-5
Risk of bias across studies	22	Present results of any assessment of risk of bias across studies (see Item 15).	Results –9th paragraph and Figure 6
Additional analysis	23	Give results of additional analyses, if done (e.g., sensitivity or subgroup analyses, meta-regression [see Item 16]).	Results –9th paragraph and Table S5
**DISCUSSION**			
Summary of evidence	24	Summarize the main findings including the strength of evidence for each main outcome; consider their relevance to key groups (e.g., healthcare providers, users, and policy makers).	Discussion – 1st to 8th paragraphs
Limitations	25	Discuss limitations at study and outcome level (e.g., risk of bias), and at review-level (e.g., incomplete retrieval of identified research, reporting bias).	Discussion – 9th paragraph
Conclusions	26	Provide a general interpretation of the results in the context of other evidence, and implications for future research.	Discussion – 10th paragraph
**FUNDING**			
Funding	27	Describe sources of funding for the systematic review and other support (e.g., supply of data); role of funders for the systematic review.	Acknowledgements

From: Moher D, Liberati A, Tetzlaff J, Altman DG, The PRISMA Group (2009). Preferred Reporting Items for Systematic Reviews and Meta-Analyses: The PRISMA Statement. PLoS Med 6(7): e1000097. doi:10.1371/journal.pmed1000097. For more information, visit: www.prisma-statement.org.

Checklist S2. STROBE Statement—Checklist of items that should be included in reports of cross-sectional studies

No	Questions	Score	
		Yes=1	No=0
**1.**	**Are the research questions clearly stated?**		
**2.**	**Is the approach appropriate for the research question?**		
**3.**	**Is the study context clearly described?**		
**4.**	**Is the role of the researcher clearly described?**		
**5.**	**Is the sampling method clearly described?**		
**6.**	**Is the sampling strategy appropriate for the research question?**		
**7.**	**Is the method of data collection clearly described?**		
**8.**	**Is the data collection method appropriate to the research question?**		
**9.**	**Is the method of analysis clearly described?**		
**10.**	**Are the main characteristics of the population well described?**		
**11.**	**Is the analysis appropriate for the research question?**		
**12.**	**Are the claims made supported by sufficient evidence?**		

Transcribed from the original paper: Moosazadeh, M., Nekoei-Moghadam, M., Emrani, Z. & Amiresmaili, M. Prevalence of unwanted pregnancy in Iran: a systematic review and meta-analysis. *The International Journal of Health Planning and Management* **29**, e277-e290 (2014). Information on the STROBE Initiative is available at www.strobe-statement.org.
